# DNA-nanostructure-templated assembly of planar and curved lipid-bilayer membranes

**DOI:** 10.3389/fchem.2022.1047874

**Published:** 2023-02-08

**Authors:** Mostafa A. Elbahnasawy, Mahmoud L. Nasr

**Affiliations:** ^1^ Immunology Laboratory, Botany and Microbiology Department, Faculty of Science, Al-Azhar University, Cairo, Egypt; ^2^ Renal Division and Engineering in Medicine Division, Department of Medicine, Brigham and Women’s Hospital, Harvard Medical School, Boston, MA, United States

**Keywords:** DNA nanostructures, liposomes, DNA-corralled nanodiscs, DNA origami, nanodiscs, membrane proteins, viral entry

## Abstract

Lipid-bilayer nanodiscs and liposomes have been developed to stabilize membrane proteins in order to study their structures and functions. Nanodiscs are detergent-free, water-soluble, and size-controlled planar phospholipid-bilayer platforms. On the other hand, liposomes are curved phospholipid-bilayer spheres with an aqueous core used as drug delivery systems and model membrane platforms for studying cellular activities. A long-standing challenge is the generation of a homogenous and monodispersed lipid-bilayer system with a very wide range of dimensions and curvatures (elongation, bending, and twisting). A DNA-origami template provides a way to control the shapes, sizes, and arrangements of lipid bilayers *via* enforcing the assembly of lipid bilayers within the cavities created by DNA nanostructures. Here, we provide a concise overview and discuss how to design planar and curved lipid-bilayer membranes by using DNA-origami nanostructures as templates. Finally, we will discuss the potential applications of DNA-origami nanostructures in the structural and functional studies of large membrane proteins and their complexes.

## 1 Introduction

Over the past four decades, DNA nanotechnology has shown tremendous growth as an outstanding approach for engineering nanoscale molecules using synthetic DNA as constructing blocks (Rothemund, 2006; Seeman, 2010; Seeman and Sleiman, 2017). As a building material, DNA molecules are considered one of the most defined, predictable, and programmable material due to their specific sequence programmability, synthesis accessibility, rigidity, self-assembly, biocompatibility, and thermodynamic stability (Chen et al., 2015; Seeman and Sleiman, 2017; Bujold et al., 2018). This flexibility in the structural design by DNA allowed the exclusively *de novo* designing of precisely defined structures in nearly any shape and size and with additional capability to control self-assembly in both static and dynamic ways (Jones et al., 2015). Several assembly methods have been developed to make DNA nanostructures, including DNA-origami (Rothemund, 2006; Douglas et al., 2009), single-stranded DNA tiles (Wei et al., 2012), supramolecular DNA assembly, and polyhedral mesh method (Benson et al., 2015). Among all these DNA assembly methods, DNA-origami has been extensively used due to its robustness and versatility for the custom building of not only 2D and 3D static nanostructures (Rothemund, 2006; Douglas et al., 2009) but also dynamic nanostructures, including nanodevices and robots (Hong et al., 2017).

Membrane proteins (MPs) are a category of proteins that are constituents of all biological membranes including the plasma membranes and membranes that envelope the intracellular organelles. Based on various predictions by multiple methods, up to 28% of the entire human protein-coding genes encode for MPs (Lander et al., 2001; Fagerberg et al., 2010; Attwood et al., 2017). The structure of MPs is versatile to provide enormous functionality to the cells. MPs function as receptors, transporters, channels, enzymes, and redox facilitators. In addition, they are pivotal players in several biological processes such as ion transport, cellular signaling, and cell adhesion (Cournia et al., 2015). Given their essential physiological roles, more than 60% of FDA-approved drugs target MPs (Overington et al., 2006). Despite their current immense medical importance and relative abundance, only the structures of a small fraction of MPs have been determined. The main challenges for structural and functional studies of MPs are their insolubility, instability, and inactivity upon isolation from their native lipid-bilayer membranes. Indeed, MPs are neither soluble nor functional without their native lipid-bilayers or lipid-bilayer mimetics (Yeagle, 2016).

Various lipid-bilayer mimetics have been developed to stabilize MPs for studying their structures and functions. These membrane mimicking systems include detergent-based platforms (micelles) and detergent-free systems (liposomes and nanodiscs). Micelles can lower the stability of MPs and hence reduce or even abolish their biological functions (Seddon et al., 2004). Additionally, detergents can interfere with the interactions between MPs and their soluble partners. Both liposomes and nanodiscs provide detergent-free phospholipid-bilayer platforms and thus save the stability and functionality of MPs. Liposomes are heterogeneous in size, and thus it is hard to control the number of copies of a given membrane protein for each particle. Nanodiscs are planar lipid-bilayer platforms with relatively homogeneous sizes and thus provide native-like environments for MP studies (Bayburt and Sligar, 2010). In this review, we discuss how to harness DNA-origami nanostructures for constructing planar and curved lipid-bilayer membranes with well-defined shapes, sizes, and arrangements. In addition, we will discuss the applications of DNA-scaffolded lipid-bilayer membranes for studying the structure and function of large MPs.

## 2 DNA origami

**FIGURE 1:**
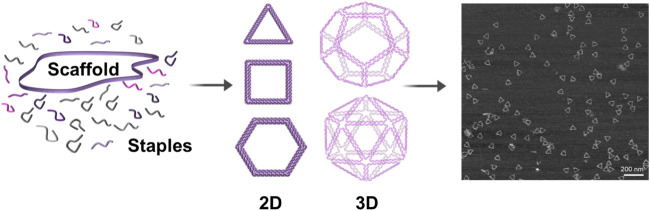
Schematic representation of DNA-origami formation. A long single-stranded DNA oligo (scaffold) is self-folded—in a single annealing step—with the help of hundreds of short single-stranded DNA oligos (staples) into any desired shape. Staples are base-paired to multiple parts of the scaffold to crosslink spatially distant segments of the scaffold together and to stabilize the folding of scaffold. Right: AFM image of the triangular DNA origami (Jun et al. 2019).

The DNA-origami technique is a one-pot reaction for self-assembly of DNA molecules in a bottom-up manner. In this technique, long single-stranded DNA (ssDNA) oligos (scaffold strands) are self-folded—in a single annealing step—with the help of hundreds of short ssDNA oligos (staple strands) into any shape of interest (Rothemund, 2006; Douglas et al., 2009), with dimensions ranging from nanometers to micrometers (Tikhomirov et al., 2017) and molecular masses up to the gigadalton scale (Wagenbauer et al., 2017) (Figure 1). Scaffold strands are usually derivatives of M13 bacteriophage genomic DNA (6407 nucleotides, circular ssDNA). Staple strands are typically 20–60 bp long and designed to be complementary to different parts of the scaffold strands to crosslink spatially distant segments of a scaffold strand together. These anti-parallel double-crossovers create thousands of base pairs to stabilize the folding of scaffolds (Dietz et al., 2009; Shih and Lin, 2010). Unlike other assembly methods, crude or unpurified DNA staple oligos can directly be used in DNA origami and higher yields are produced in just a few hours of annealing; therefore, time and effort can be saved. Furthermore, DNA origami has exclusively allowed the construction of more complex shapes. Given the capability to control both the direction and bending degree in a precise manner, DNA origami has been used extensively for the fabrication of complicated and unprecedented nanoscale architectures, including nanostructures with high twists and curvatures (Dietz et al., 2009; Han et al., 2011).

Several architectures of DNA origami were successfully invented to arrange a variety of nanomaterials (nanoparticles, carbon nanotubes, nanorods, quantum dots, and liposomes) with predetermined numbers, positions, and directions of nanomaterials, resulting in unprecedented DNA-origami–nanomaterial complexes with novel properties. Inspired by DNA-origami advantages, our group designed DNA-origami barrels for serving as scaffolding corrals to make and stabilize large-sized nanodiscs (Zhao et al., 2018).

## 3 DNA lipid-bilayer conjugation methods

**FIGURE 2:**
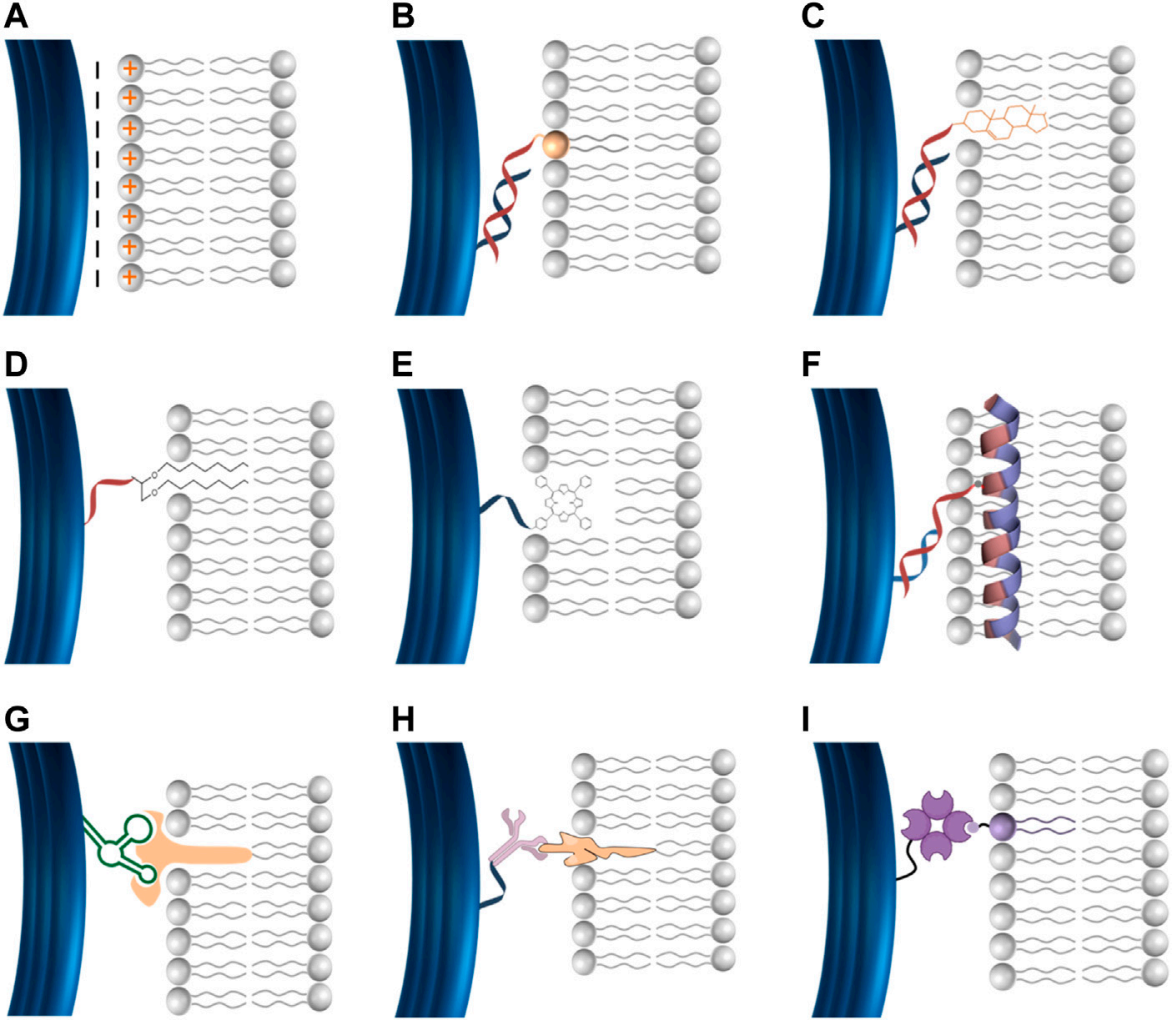
DNA lipid-bilayer membranes conjugation methods. **(A)** DNA can electrostatically bind to cationic lipid bilayers. DNA can bind to lipid bilayers *via* hydrophobic moieties, including phospholipids **(B)**, cholesterol **(C)**, ethyl–thiol groups **(D)**, and porphyrin **(E)**. DNA can bind to lipid bilayers *via* amphipathic molecules such as the membrane scaffold proteins (MSPs) **(F)**. Specific membrane-associated molecules for a ligand–receptor interaction, including aptamer–target **(G)**, antigen–antibody **(H)**, and biotin–streptavidin **(I)** can be used to facilitate the conjugation of DNA to lipid-bilayer membranes.

Current approaches have been reported for the conjugation of DNA to lipid-bilayer include electrostatic, hydrophobic, and ligand-based interactions (Figure 2). For electrostatic-based conjugation, DNA nanostructures (negatively charged due to the phosphate backbones) can be electrostatically attracted onto lipids surfaces of either cationic (positive) or zwitterionic (net neutral) charged headgroups (Figure 2A) (Suzuki et al., 2015). The interaction between DNA and lipids can be mediated by divalent cations, such as Mg^2+^, Ca^2+^, and others (typically found in the origami folding buffer), which work as salt bridges between lipids and DNA (Gromelski and Brezesinski, 2006; Mengistu et al., 2009). One benefit of this conjugation strategy is that DNA nanostructures remain mobile on the surface of lipid bilayers, which enable the formation of extended ordered superstructures (micrometer) on the bilayer surface (Suzuki et al., 2015).

To stabilize and strengthen the binding between DNA and lipid-bilayer membranes, hydrophobic moieties can be used to facilitate robust conjugation (Figures 2B–E). DNA can be covalently functionalized with hydrophobic motifs, including cholesterol anchor, phospholipids, ethyl–thiol groups, α-tocopherol, poly (propylene oxide), and porphyrin (Langecker et al., 2014; Iwabuchi et al., 2021). Cholesterol is the hydrophobic anchor most commonly used to conjugate DNA, resulting in irreversible binding with lipid-bilayer membranes (Langecker et al., 2014). Thus, multiple cholesterol modifications on DNA would strengthen the conjugation to biological membranes. Cholesterol is usually linked to tetra-(ethylene glycol) to avoid any potential alterations on the membrane structure, a possibility if cholesterol is used alone (Langecker et al., 2014). Additionally, DNA can bind to the lipid bilayer *via* intermediate amphipathic molecules such as the membrane scaffold proteins (MSPs) that are usually used to assemble conventional nanodiscs. We have designed versions of MSPs with cysteine mutants to provide the ability for covalent coupling to DNA oligo upon treatment with Sulfo-SMCC crosslinkers (F) (Figure 2F) (Zhao et al., 2018).

For targeted binding and enhanced specificity in the conjugation of DNA to lipid-bilayer membranes, specific membrane-associated molecules, including antigen–antibody, aptamer–target, biotin–streptavidin, and covalent interactions can be used. DNA nanostructures can be decorated by ligands to interact with their receptors inserted into the lipid bilayer to facilitate precise conjugation (Figures 2G–I). Regardless of the high specificity of this conjugation method, its application is limited to some specific circumstances due to their reversibility and difficulty to control the stoichiometric ratios which affect the stability of DNA lipid bilayer, restricting their application in the physiological environment. Overall, oligo-based conjugation has been introduced for a more precise, robust, and stable DNA binding to lipid-bilayer membranes. DNA nanostructures can be decorated with ssDNA (handle) to bind to the complementary ssDNA (anti-handle) coupled to lipid-bilayer membranes (Yang et al., 2016; Zhang et al., 2017; Bian et al., 2019).

Further studies are needed to investigate the behaviors of the incorporated proteins and lipids within the DNA nanostructures.

## 4 DNA origami facilitates curved lipid-bilayer membrane assembly

Liposomes are synthetic spherical vesicles of amphiphilic bilayer lipids (often phospholipids) with an aqueous core. They are important drug delivery systems and model membrane platforms for studying cellular activities (Elbahnasawy et al., 2018; Wang et al., 2018; Xu et al., 2021). Traditional approaches can construct liposomes with certain sizes and considerable homogeneity, but the generation of monodispersed liposomes with a diverse range of dimensions, shapes, lipid compositions, and defined nanoscale geometry is still a big challenge. With the recent ability to tune the shape of DNA origami in any dimensions and curvatures (elongation, bending, and twisting) (Wang et al., 2021), it would mimic cellular membrane dynamics to provide more opportunities for the structural and functional study of curvature-dependent cellular activities.

**FIGURE 3:**
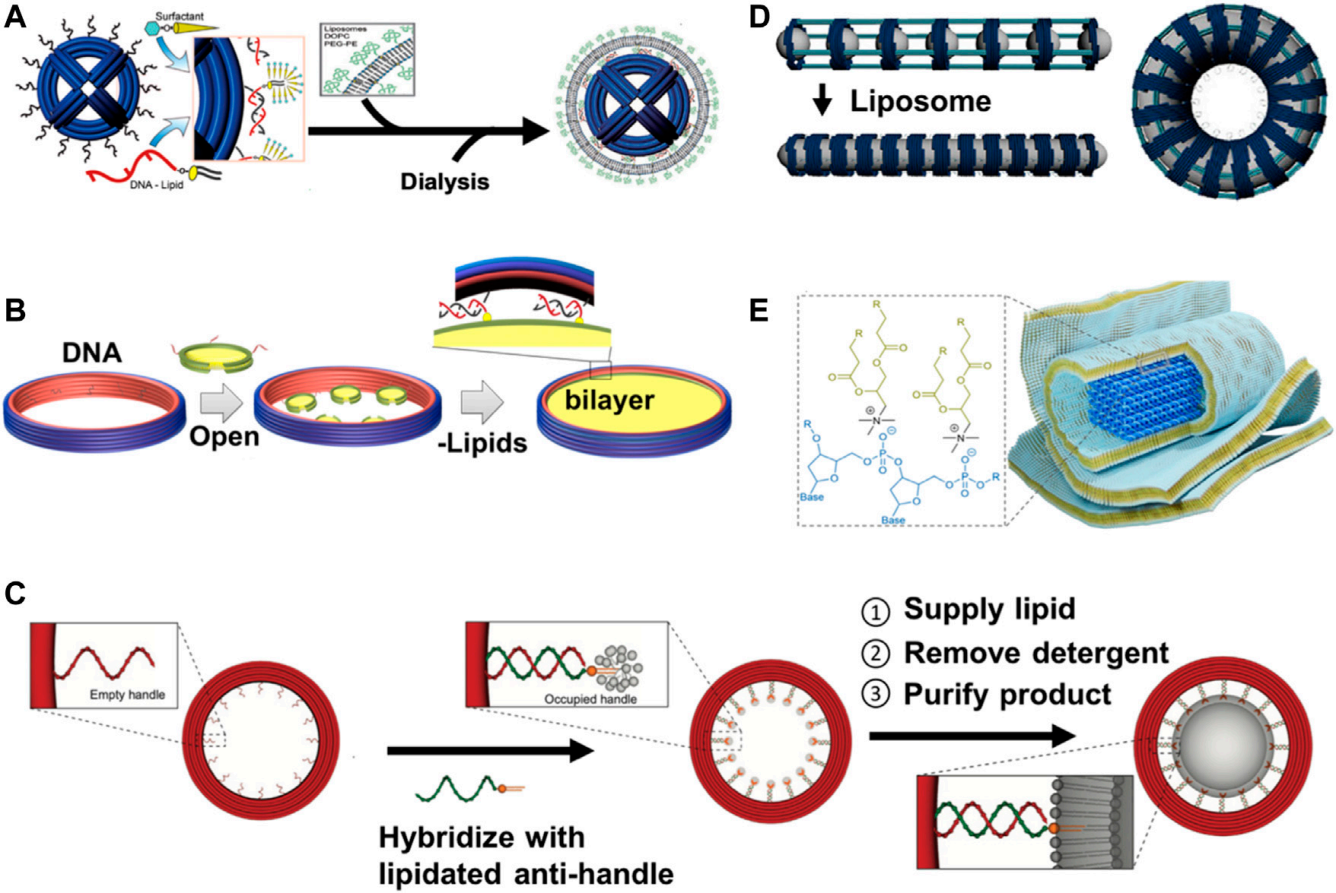
Schematic illustration of DNA nanostructures templated the formation of curved (liposomes) and planar (nanodiscs) lipid-bilayer membranes. **(A)** DNA-origami spheres can be used as inner (endoskeleton) templates for constructing liposomes around spheres *via* outer DNA handles annealed to anti-handle lipid conjugates (figure used with permission from Perrault and Shih 2014). **(B)** DNA-origami barrels can be first decorated by small nanodiscs (oligo-conjugated) *via* DNA hybridization, incubated with excess liposomes and detergents, dialyzed, and finally purified by sucrose-gradient centrifugation to reconstitute large-sized DNA-corralled nanodiscs (figure used with permission from Zhao et al., 2018). **(C)** DNA-origami rings can be used as outer (exoskeleton) templates for forming size-controlled liposomes inside the rings through handle and anti-handle assembly (figure used with permission from Yang et al., 2016). **(D)** DNA-origami nanocages were externally templated for the formation of shape-defined liposomes arranged in arrays to make width-defined membrane tubules and toroidal liposomes to mimic lipid-bilayer membrane curvatures (elongation, bending, and twisting) present in the cells (figure used with permission from Zhang et al., 2017). **(E)** DNA-origami helix bundle can be used as endoskeleton templates for growing of multilamellar lipid structures through electrostatic interactions between the positively charged headgroup of the lipid molecules and the negatively charged phosphate groups in the DNA backbone (figure used with permission from Julin et al., 2020).

Scaffolded DNA-origami nanostructures have been proven to be outstanding templates for constructing geometrically defined liposomes. Perrault and Shih (2014) designed octahedron-shaped DNA origami as inner (endoskeleton) templates for the formation of liposomes around them (Figure 3A) (Perrault and Shih, 2014). Yang et al. (2016) used scaffolded DNA origami as external structure molds to guide liposome formation inside these rigid DNA-origami spheres. The interior surface of DNA-origami rings was decorated with docking oligo strands (handle) that enabled them to hybridize with lipid-modified oligo strands (anti-handle) for the formation of liposome and to control their sizes and shapes (Figure 3C) (Yang et al., 2016). Lin and coworkers designed DNA origami to organize well-defined (size and shape) liposomes at specific distances for studying the lipid transfer. In their preparation, DNA-origami rings were lined by 32 ssDNA handles to hybridize to lipidated anti-handles to nucleate the formation of liposomes within the rings. The rings were held by DNA pillars at a well-defined distance (Bian et al., 2019). Zhang et al. (2017) reported well-controlled liposome formation within nanocages of DNA nanostructures, (Figure 3D) (Zhang et al., 2017). Another recent application of DNA origami as endoskeleton templates was carried out by Kostiainen and colleagues (2020). They designed DNA-origami helix bundles as inner templates and nucleation sites for growing multilamellar lipid structures through electrostatic interactions between the lipid molecules (positively charged headgroups) and DNA backbones (negatively charged phosphate groups) (Figure 3E) (Julin et al., 2020).

## 5 DNA origami facilitates planar lipid-bilayer membrane assembly

### 5.1 Nanodiscs and size matter

Nanodiscs are planar lipid-bilayer mimetics consisting of disc-shaped structures wrapped by two copies of an amphipathic alpha-helical protein, called membrane scaffold proteins (MSPs) (Bayburt et al., 2002). The MSPs are genetically engineered forms of human serum apolipoprotein A1 (ApoA1), which is the major protein of high-density lipoprotein (HDL) (Bayburt et al., 2002; Ritchie et al., 2009). The MSPs were designed to self-assemble around lipid-bilayer patches, thus enclosing, shaping, and stabilizing nanodiscs. Any kind of bilayer-forming lipids can be used to form the nanodiscs (Ritchie et al., 2009). As a detergent-free lipid-bilayer model, nanodiscs have provided robust means for keeping membrane protein targets soluble, while providing a physiologically relevant and native-like bilayer environment that maintains their functional activity (Bayburt et al., 1998). With the ability to offer a very convenient, adjustable-in-size, and lipid composition-bilayer environment, nanodiscs have evoked significant interest as a versatile platform for studying all types of MPs using NMR and cryo-EM. A tremendous number of MPs have been successfully incorporated into nanodiscs, including human mitochondrial voltage-dependent anion channel (hVDAC-1) (Nasr et al., 2017), human TRPM4 ion channel (Autzen et al., 2018), human mitochondrial Bax protein (López et al., 2019), cytochrome b5 (Bai et al., 2020), mouse volume-regulated anion channel LRRC8 (Kern et al., 2019), HIV-1 envelope glycoprotein (Wang et al., 2019; Elbahnasawy et al., 2020), bacterial outer-membrane protein OmpX (Hagn et al., 2013), bacteriorhodopsin (Kooijman et al., 2019), and *Drosophila* odorant receptors (Murugathas et al., 2019).

Nanodiscs surpass all recent membrane-mimicking systems (micelles, bicelles, amphipols, and liposomes) by providing detergent-free, soluble, stable, small, monodispersed, and size-homogenous lipid-bilayer platforms. These advantages would give MPs more native-like environments for their structural and functional studies (Bayburt and Sligar, 2010). The very adjustable thickness of the phospholipid bilayer is another important advantage offered by nanodiscs, which would be beneficial for MPs that either need more or less expanse of the bilayer region or undergo relative movements for interactions (Puthenveetil et al., 2017). In addition, nanodiscs can control the oligomerization of the targeted MPs by changing the ratios of MSPs to lipid to membrane proteins (Bayburt and Sligar, 2010; Nasr et al., 2017). Another benefit of nanodiscs is that both sides of the nanodiscs are accessible (Banerjee et al., 2008; Ritchie et al., 2009). However, the size of conventional nanodiscs is restricted to less than 16 nm in diameter (Denisov and Sligar, 2017), which limits their use to studying large MPs and their complexes. In addition, extending the size of nanodiscs would also be useful to study the early viral entry steps, including virus–host membrane fusion and genome translocation (Nasr et al., 2017; Nasr and Wagner 2018). To this end, we have engineered covalently circularized nanodiscs (cNDs) with a wide range of diameters (∼8, 11, 15, and 50 nm) and defined geometric shapes, made by circularized MSPs (Nasr et al., 2017). These circularized MSPs were designed to have a recognition sequence for sortase A (LPGTG) at C terminus and a single glycine (G) residue at N terminus, thus circularized *via* the formation of a covalent linkage between the N and C termini upon treatment with sortase A. Compared to conventional nanodiscs, cNDs demonstrated remarkable homogeneity in size and shape and enhanced thermal stability. These advantages facilitate cND utility in solution NMR and single-particle cryo-EM structural determination. Ascribed to the wide scale of diameter provided by cNDs (8–50 nm), cNDs have attracted a significant amount of interest as a versatile membrane model to study both small- and medium-sized MPs and their macromolecular complexes (Bao et al., 2018; McGoldrick et al., 2018). Moreover, cNDs can be used as a surrogate membrane for studying the earlier interactions between small viruses with their receptors (Nasr et al., 2017; Nasr and Wagner 2018). Therefore, constructing larger cNDs is of high value not only for the reconstitution of larger membrane protein complexes but also to study the early steps of cell entry for relatively large viruses, e.g., influenza (80–120 nm), HIV-1 (120 nm), herpes simplex virus 1 (125 nm), Epstein–Barr virus (120–180 nm), and coronaviruses (50–200 nm). Approaches toward producing cNDs bigger than 50 nm in diameter have proven to be difficult and elusive due to their instability and propensity to aggregate in larger sizes. Moreover, the expression of sufficiently long MSPs for larger nanodiscs has proven to be difficult in the *E. coli* expression system (Zhao et al., 2018).

### 5.2 DNA origami enables the assembly of large-sized nanodiscs

Engineering large-sized nanodiscs with a prescribed geometry is a daunting challenge. Building large-sized nanodiscs (over 50 nm in diameter) needs a shielding template as a scaffolding constrainer, rather than the MSP, to protect large nanodiscs from aggregation. An ideal scaffolding template should be easy for manipulation (controlling its shape and size). DNA origami has been used in so many fields as a versatile method for the bottom-up fabrication of novel nanoscale materials and devices with well-defined shapes and sizes (Rothemund, 2006; Douglas et al., 2009). In addition, the atomic precise spatial addressability, predictability, and high rigidity of DNA origami have made them scaffolds for anchoring a variety of functional ligands (e.g., DNA, peptides, proteins, aptamers, fluorophores, nanoparticles, and biomolecules) with a near-angstrom organization of these ligands on the nanostructures (Castro et al., 2011; Pei et al., 2013). Inspired by these unique capabilities, we conceptualized that DNA origami could be used as a bumper case to stabilize large nanodiscs. To this end, a closed ribbon- or barrel-shaped DNA origami was designed to externally support the growth of lipid-bilayer membrane and encircle the assembled nanodiscs.

The software caDNAno was used to design and assemble the 3D barrel-shaped DNA origami with the desired geometry and prescribed dimensions. Then, DNA-origami nanobarrels was folded with a 10-fold excess of staples using a thermal (25°C–65°C) annealing program for > 20 h. The properly folded DNA barrels were purified by glycerol-gradient centrifugation (15%–45%) and confirmed by negative-stain transmission electron microscopy (TEM) and native agarose gel electrophoresis. Following this procedure, 60-nm and 90-nm diameter barrel-shaped DNA origami were designed to construct nanodiscs with ∼45 nm and ∼70 nm in diameter, respectively (Zhao et al., 2018).

#### 5.2.1 Small nanodisc-decorated DNA-barrel assembly

DNA origami was decorated with conventional, 11-nm nanodiscs at its inner face. The addition of detergents and lipids (in the form of liposomes) to small nanodisc-decorated barrels followed by dialysis resulted in the reconstitution of large nanodiscs inside the cavity of the barrels. Standard procedures for the generation of nanodiscs scaffolded by DNA origami can be summarized as follows: designing of barrel-shaped DNA-origami templates with extending handles for subsequent lipid/protein binding, designing of oligo-conjugated small nanodiscs, hybridizing the anti-handle (complementary ssDNA) in small nanodiscs to the DNA-origami templates, adding an excess of lipids, and finally the purification of the scaffolding phospholipid-bilayer DNA origami. DNA-origami barrels were designed with several handles (36 and 24 handles for 90-nm and 60-nm DNA origami, respectively) for subsequent hybridization with the anti-handle linkers that are covalently coupled to MSPs of nanodiscs. The MSP of small nanodiscs was mutated to insert three cysteines to facilitate the covalent coupling of the anti-handles through Sulfo-SMCC crosslinkers. The anti-handle-modified nanodiscs were allowed to hybridize to the handle-decorated DNA-origami templates as illustrated in Figure 3B.

#### 5.2.2 The assembly of large interconnected bilayer within DNA barrels

Sequential steps should be taken with care for the self-assembly of large nanodiscs from their small-nanodisc precursor. The numbers of initial small nanodiscs are dependent on the diameter of DNA-origami barrel; larger diameter DNA barrels require more nanodiscs. Enough nanodiscs should be assembled and hybridized to the inner side of each DNA barrel, leaving only a few nanometers between each nanodisc. Once these nanodiscs come close to each other, they are now ready for merging to make the large bilayer. The addition of detergents and lipids destabilizes the small nanodiscs and allows for the merging of the neighboring ones to reconstitute DNA-corralled nanodiscs. For this reason, we used the linear MSPs, not the circularized one, in the initial steps. Finally, the assembled DNA-corralled nanodiscs (DCNDs) were purified by a density (sucrose)-gradient ultracentrifugation to remove any excess lipid molecules.

#### 5.2.3 Stability of DNA-corralled nanodiscs (DCNDs)

Maintaining the structural integrity and stability of the DCNDs are required for using them as platforms to study the functions and structures of MPs. It is well established that DNA-origami assembly is dependent on buffer conditions. For assembly, DNA origami needs TAE buffer with Mg^2+^ in the mM range. However, such high Mg^2+^ concentrations are not critical for ensuring DNA-origami stability, as these assembled DNA origami still maintain their structural integrity even after transferring into buffers with low-μM concentrations of Mg^2+^ (Ramakrishnan et al., 2018; Wang et al., 2015). However, it was expected that DNA origami, under low Mg^2+^ concentrations, would be prone to denaturation caused by potential interactions of Mg^2+^ ions with other buffer components which eventually results in reducing the Mg^2+^ role in electrostatic repulsion within the DNA-origami construct. DCNDs revealed good stability in Mg^2+^-free buffers containing 50 mM Ca^2+^ or 10 mM K^+^, while DNA-origami nanotubes denatured in Mg^2+^-free buffers containing 200-mM Ca^2+^ or K^+^ (Wang et al., 2015).

DCNDs exhibited a good tolerance in a broad range of pH from 4.0 up to 10.0. Similarly, DNA-origami nanotubes also showed stability at pH range 5.0–10.0 (Wang et al., 2015). Moreover, DCNDs revealed long-term stability of up to 40 days at 4°C. Most remarkably, DNA-origami nanostructures have shown stability with denaturants like urea or guanidinium chloride, which are widely used in biophysical and biochemical studies (Ramakrishnan et al., 2017).

### 5.3 Potential applications of DCNDs

#### 5.3.1 Virus entry

**FIGURE 4:**
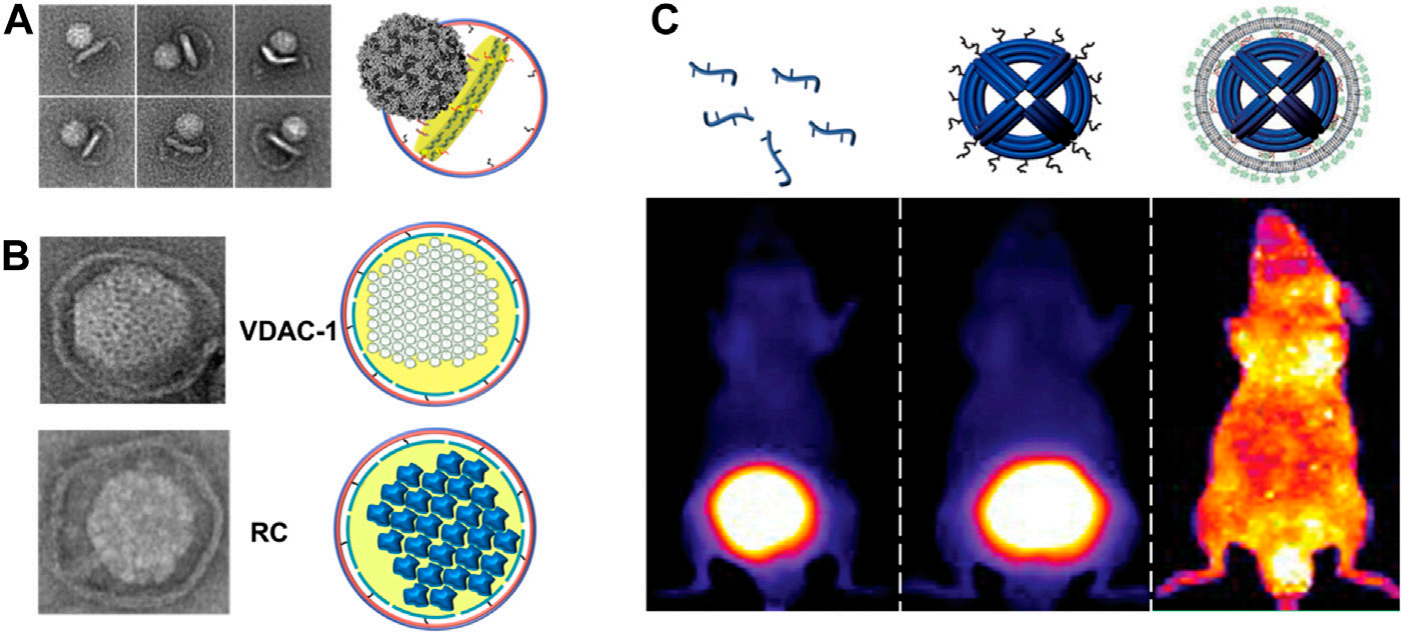
**(A)** Binding and interactions of poliovirus with *CD155*-decorated DNA-corralled nanodiscs (DCNDs). TEM images showing individual viral particles tethered to DCNDs. Some images show the bending of the bilayer by the poliovirus (figure used with permission from Zhao et al., 2018). **(B)** DCNDs were used to reconstitute human voltage-dependent anion channel 1 (hVDAC1) and *Rhodobacter sphaeroides* photosynthetic reaction center (RC) protein clusters (figure used with permission from Zhao et al., 2018). **(C)** Encapsulation of DNA origami within lipid bilayers achieves better *in vivo* stability (protection against nucleases digestion) and bio-distribution. Fluorescent images of mice showing the bio-distribution of Alexa-Fluor750-labeled oligonucleotide, DNA origami, and encapsulated DNA origami inside lipid bilayers (figure used with permission from Perrault and Shih 2014).

Figure 4 shows some applications of DNA origami, such as virus entry, reconstitution of MPs, and *in vivo* delivery applications. Harnessing DCNDs as a surrogate membrane for studying the early steps involved in virus infection, in our opinion, is one of the novel applications offered by large-sized nanodiscs. We earlier speculated that extending the diameter of nanodiscs would be useful in studying the viral entry of both larger non-enveloped and enveloped viruses (Denisov et al., 2004; Nasr and Wagner, 2018). DCNDs have been used to study the early steps involved in poliovirus entry. Poliovirus, the etiologic agent of polio or poliomyelitis, is a simple non-enveloped virus composed of (+) ssRNA with ∼7500-nucleotide-long genome enclosed by a protein icosahedral-shaped capsid (∼30 nm in diameter) (Hogle, 2002; Tuthill et al., 2010). The attachment of poliovirus to its receptor *CD155* will subsequently catalyze conformational rearrangements and expansion of the virus particle (Mendelsohn et al., 1989), which ends up by injection of RNA across the membrane. We have prepared and used 45-nm DCNDs decorated with the poliovirus receptor *CD155* to study the early steps for poliovirus entry and genome translocation. Negative-stain TEM confirmed the binding of the poliovirus to CD155-modified DCNDs and the subsequent formation of pores into nanodiscs (Figure 4A). This observation is consistent with our previous data (Nasr et al., 2017), where poliovirus particles interacted with *CD155*-decorated 50-nm cNDs and made a putative pore in cNDs for ejecting their RNA across the bilayer membranes.

#### 5.3.2 Human voltage-dependent anion channel 1 (hVDAC1) protein

As the most abundant protein within the mitochondrial outer membrane (MOM), the hVDAC1 is the primary gatekeeper of mitochondria, interlinking the cytosolic and mitochondrial compartments and regulating the metabolic flux across the mitochondrial outer membrane. This channel protein undergoes homo or hetero oligomerization, releasing cytochrome c to the cytoplasm and leading to mitochondrially induced apoptosis. Therefore, its expression level contributes to the phenotype of cancer cells (Bayrhuber et al., 2008; Shoshan-Barmatz and Mizrachi, 2012; Hosaka et al., 2017). Moreover, hVDAC1 also serves as a binding point for mitochondria-interacting proteins, such as hexokinase, B-cell lymphoma 2 (Bcl-2), and B-cell lymphoma-extra large (Bcl-xL) (Shoshan-Barmatz and Mizrachi, 2012).

To assemble hVDAC1 into DCNDs, detergent-solubilized hVDAC1 were added along with lipids, followed by dialysis then purification using a density-gradient ultracentrifugation. Furthermore, the incorporation of hVDAC1 into DCNDs was verified for the incorporation of hVDAC1 by using negative-stain TEM and SDS-PAGE (Figure 4B). The mitochondrial membranes also contain a unique phospholipid called cardiolipin as a basic component. So, future studies should include cardiolipin in DCNDs for studying the assembly of hVDAC1 on the mitochondrial membrane surface.

#### 5.3.3 *Rhodobacter sphaeroides* photosynthetic reaction center (RC) protein

The RC acts as a photo-converter of light into chemical energy and it was first isolated from the purple bacterium *Rhodobacter sphaeroides*. The RC is a transmembrane protein complex, consisting of four bacteriochlorophylls, that catalyzes light-driven electron transport across photosynthetic membranes. The RC is mostly an *α*-helical protein and binds nine cofactors to form two potential electron-transfer chains (Ermler et al., 1994; Lin et al., 2009). The RC not only harvests light and photo-generates charges but also stores them, realizing these photo-proteins as self-charging bio-photonic devices (Ravi et al., 2019). Following the aforementioned procedure for the incorporation of hVDAC1 into DCNDs, RC was assembled and verified for incorporation by negative-stain TEM and SDS-PAGE (Figure 4B). DCNDs provide a novel approach for designing nanodisc-based solar nano-machines by the assembling/disassembling of solar energy conversion complexes. An earlier study demonstrated the incorporation of RC into DMPC 10-nm nanodiscs arranged in parallel on the surface of a carbon nanotube (Ham et al., 2010).

## 6 Future directions

**FIGURE 5:**
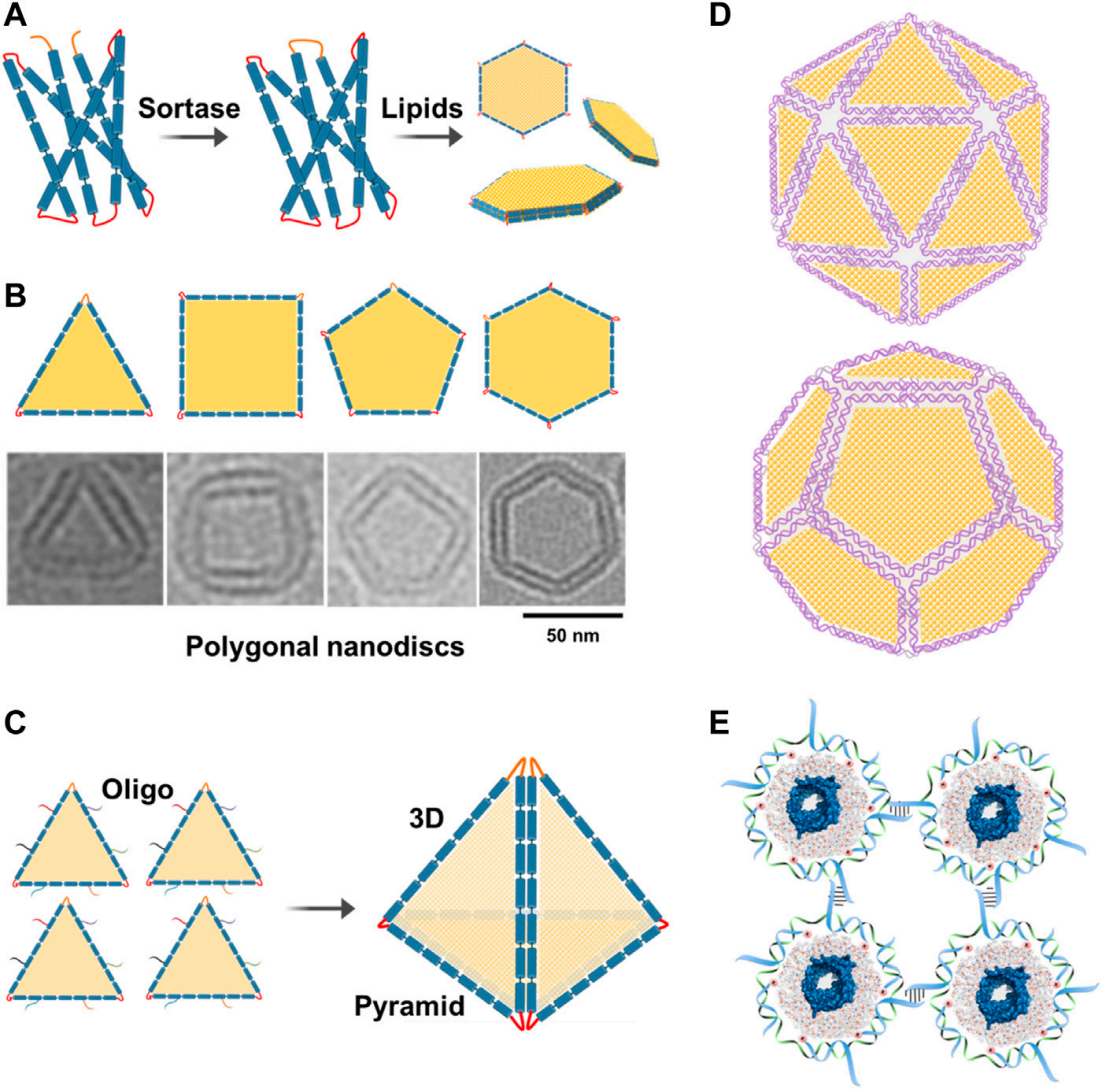
Future directions. **(A)** Assembly of polygonal nanodiscs. Large circularizable membrane scaffold proteins can be engineered to allow the assembly of well-defined polygonal shapes such as hexagonal nanodiscs. **(B)** Cryo-EM images of individual nanodiscs with different shapes. Adding more lipids to these nanodiscs can change their shapes back to circular nanodiscs. **(C)** Polygonal nanodiscs can be used to assemble 3D structures that can be used in drug or gene delivery. For example, four triangular nanodiscs can be stitched together with the help of complementary oligos to assemble a pyramid which can be used in the delivery of drugs or genes. **(D)** Polygonal DNA nanodiscs can be used to assemble 3D structures of well-defined sizes and shapes similar to viruses. Additionally, these particles can be further modified with ligands or proteins on their surfaces. **(E)** DNA-corralled or DNA-belted nanodiscs containing membrane proteins can be used to form 2D lattice or functional materials.

DNA-supported lipid bilayers are expected to find applications in diverse areas ranging from basic research to synthetic biology. DNA-nanostructure-templated lipid-bilayers provide unique platforms that can recapitulate the intrinsic tension and curvature in biological membranes, thus facilitating the study of topology and functions of their associated membrane proteins. DNA-corralled nanodiscs have shown the potential for revealing the early steps of host and microbe interactions which could solve outstanding questions including genome translocation and help define the structural changes associated with cell entry at an unprecedented level of details. Inspired by DNA origami, large circularized scaffold proteins can be engineered to allow the assembly of well-defined 2D and potentially 3D polygonal shapes, such as 2D hexagon-shaped nanodiscs and 3D pyramid-shaped nanostructures, respectively, for the delivery of drugs or genes (Figures 5A–C). This ability to precisely fold circularized scaffold proteins into different shapes inspired us to further engineer them and create large protein scaffolds that can be folded into well-defined shapes similar to the M13 DNA scaffold. To our knowledge, this is the first example of folding a large membrane scaffold proteins into well-defined polygonal shapes. Herein, we would like to introduce the new concept of “protein origami”. Furthermore, polygonal nanodiscs can be used to assemble 3D structures of well-defined sizes and shapes similar to viruses. Additionally, these virus-like particles can be further modified with ligands or proteins on their surfaces which expand their applications as innovative vaccine platforms (Figure 5D).

DNA-corralled or DNA-templated nanodiscs containing membrane proteins can be used to form 2D lattice or functional materials that could be useful in different nanotechnological applications (Figure 5E).

Styrene–maleic acid (SMA) copolymers have been used successfully to extract membrane proteins from their native biological membranes without detergents (Orekhov et al., 2019). Therefore, replacing MSP in DNA-corralled nanodiscs by SMA copolymers may result in a larger DNA–SMA scaffold that is able to isolate membrane proteins and their complexes from native environments without the use of any detergents. Cellular membranes have a very wide variety of shapes, including tubules, folds, pores, and vesicles, to support their corresponding functions. To this end, with the precise control of both directionality and bending degree, DNA origami can support the assembly of curved membranes with more complicated geometries and enable double lipid-bilayer membranes assembly such as those found in mitochondria and nucleus.
